# Rolapitant for the prevention of nausea in patients receiving highly or moderately emetogenic chemotherapy

**DOI:** 10.1002/cam4.1560

**Published:** 2018-05-23

**Authors:** Rudolph M. Navari, Bernardo L. Rapoport, Dan Powers, Sujata Arora, Rebecca Clark‐Snow

**Affiliations:** ^1^ University of Alabama Birmingham School of Medicine Birmingham AL USA; ^2^ Medical Oncology Centre of Rosebank Johannesburg South Africa; ^3^ Department of Immunology Faculty of Health Science University of Pretoria Pretoria South Africa; ^4^ TESARO, Inc. Waltham MA USA; ^5^ University of Kansas Cancer Center Westwood KS USA

**Keywords:** anthracycline/cyclophosphamide, carboplatin, chemotherapy‐induced nausea and vomiting, cisplatin, highly emetogenic chemotherapy, moderately emetogenic chemotherapy, nausea, neurokinin‐1 receptor antagonist, rolapitant

## Abstract

Most patients receiving highly or moderately emetogenic chemotherapy experience chemotherapy‐induced nausea and vomiting without antiemetic prophylaxis. While neurokinin‐1 receptor antagonists (NK‐1RAs) effectively prevent emesis, their ability to prevent nausea has not been established. We evaluated the efficacy of the long‐acting NK‐1RA rolapitant in preventing chemotherapy‐induced nausea using post hoc analyses of data from 3 phase 3 trials. Patients were randomized to receive 180 mg oral rolapitant or placebo approximately 1‐2 hours before chemotherapy in combination with a 5‐hydroxytryptamine type 3 RA and dexamethasone. Nausea was assessed by visual analog scale during the acute (≤24 hours), delayed (>24‐120 hours), and overall (0‐120 hours) phases. Post hoc analyses by treatment group (rolapitant vs control) were performed on pooled data within patient subgroups receiving cisplatin‐based, carboplatin‐based, or anthracycline/cyclophosphamide (AC)‐based chemotherapy. In the cisplatin‐based chemotherapy group, significantly more patients receiving rolapitant than control reported no nausea (NN) in the overall (52.3% vs 41.7% [*P *<* *.001]; absolute benefit [AB] = 10.6%), delayed (55.7% vs 44.3% [*P *<* *.001]; AB = 11.4%), and acute (70.5% vs 64.3% [*P *=* *.030]; AB = 6.2%) phases. Similar results were observed in the carboplatin‐based chemotherapy group, with significantly more patients receiving rolapitant than control reporting NN in the overall (62.5% vs 51.2% [*P *=* *.023]; AB = 11.3%) and delayed (64.1% vs 53.6% [*P *=* *.034]; AB = 10.5%) phases. In the AC‐based chemotherapy group, patients receiving rolapitant or control reported similar NN rates during the overall and delayed phases. Rolapitant effectively prevents nausea during the overall and delayed phases in patients receiving cisplatin‐ or carboplatin‐based chemotherapy.

## 1 INTRODUCTION

Chemotherapy‐induced nausea and vomiting (CINV) is one of the most serious treatment side effects in patients with cancer^1^, ^2^ and substantially compromises patients’ quality of life (QoL).^3^, ^4^, ^5^ The likelihood of CINV is primarily dictated by the emetogenic potential of the chemotherapy administered. In the absence of CINV prophylaxis, highly emetogenic chemotherapy (HEC), such as cisplatin, anthracycline plus cyclophosphamide (AC), or carboplatin area under the curve (AUC) ≥4 mg/mL per minute, induces emesis in >90% of patients, whereas moderately emetogenic chemotherapy (MEC), such as carboplatin AUC <4 mg/mL per minute, cyclophosphamide ≤1500 mg/m^2^, or irinotecan, causes emesis in 30% to 90% of patients.^1^, ^6^ Patient risk factors, including female sex, young age, and anticipation of nausea and vomiting, increase the probability of CINV.^1^

The acute phase (≤24 hours after chemotherapy administration) of CINV is primarily mediated by 5‐hydroxytryptamine type 3 (5‐HT_3_) receptor signaling, whereas the delayed phase (>24‐120 hours) is primarily mediated by neurokinin‐1 (NK‐1) receptor signaling.^1^ Clinical practice guidelines recommend a prophylactic triple antiemetic regimen of an NK‐1 receptor antagonist (NK‐1RA), a 5‐HT_3_ RA, and dexamethasone for patients administered cisplatin, AC, carboplatin‐based chemotherapy, or any other highly emetogenic regimen.^1^, ^6^, ^7^, ^8^, ^9^ A systematic review of randomized clinical trials showed that addition of an NK‐1RA to a regimen of a 5‐HT_3_ RA and dexamethasone improved rates of emesis in the acute, delayed, and overall (0‐120 hour) phases.^10^ The control of nausea, however, remains an unmet need, as 5‐HT_3_ RAs alone fail to control delayed nausea.^11^, ^12^ The NK‐1RAs aprepitant and netupitant have reported inconsistent results.^13^

The primary endpoint of many clinical trials evaluating CINV is complete response (CR), defined as no emesis and no use of rescue medication (RM; ie, antiemetics used after chemotherapy administration).^14^, ^15^ This endpoint may not reflect the experience of patients receiving chemotherapy,^16^ as patients may experience nausea without emesis or vice versa.^11^, ^12^ Although nausea has a greater impact than vomiting on patients’ QoL,^5^ nausea prevention is not always assessed in clinical trials as an endpoint for treatment, possibly because it is more difficult to quantify than the more objective CR endpoint. Historically, studies have assessed CR and emesis with levels of nausea severity based on patient self‐reports quantified along a visual analog scale (VAS).^17^ No significant nausea (NSN) or no nausea (NN) is defined by predefined cutoffs along this scale (<5 mm for NN and <25 mm for NSN), and the incidence of both endpoints is typically reported.^11^, ^12^, ^18^, ^19^ The wider interval that characterizes “no significant nausea” on the VAS (between 0 and 25 mm) compared with “no nausea” (0‐5 mm) has more subjectivity in measuring NSN; therefore, NN is the more objective nausea endpoint.

Rolapitant (Varubi^®^, TESARO, Inc.), a selective NK‐1RA with a half‐life of approximately 7 days, was approved in 2015 by the US Food and Drug Administration in combination with other antiemetic agents in adults for the prevention of delayed nausea and vomiting associated with initial and repeat courses of emetogenic chemotherapy.^20^ In 3 global, randomized phase 3 trials, a single 180‐mg dose of rolapitant administered prior to chemotherapy in combination with a 5‐HT_3_ RA and dexamethasone significantly improved CINV protection during the delayed phase compared with a 5‐HT_3_ RA and dexamethasone alone as measured by CR in approximately 2500 patients.^18^, ^19^ To specifically evaluate the efficacy of rolapitant for control of chemotherapy‐induced nausea, we analyzed the rates in NSN and NN and durations of nausea and significant nausea over the entire 5‐day at‐risk period for patients administered cisplatin, AC, or carboplatin‐based chemotherapy, using pooled data from 3 randomized phase 3 trials of rolapitant.^18^, ^19^

## 2 METHODS

### 2.1 Study design and patients

Design details of the 3 global, randomized, double‐blind, phase 3 studies (HEC‐1, HEC‐2, and MEC) have previously been described.^18^, ^19^ Briefly, patients were stratified by sex and randomized (1:1) to receive either 180 mg oral rolapitant or matched placebo. All patients received a 5‐HT_3_ RA and dexamethasone (active control). The trials were conducted in accordance with the Declaration of Helsinki and the International Conference on Harmonisation and Good Clinical Practice (GCP) guidelines and are registered with ClinicalTrials.gov (identifiers: HEC‐1, NCT01499849; HEC‐2, NCT01500213; MEC, NCT01500226). Eligible patients were ≥18 years of age, with a Karnofsky performance score ≥60, a predicted life expectancy of ≥4 months, and adequate bone marrow, kidney, and liver function.^18^, ^19^ For the HEC studies, patients were required to be naive to cisplatin and scheduled to receive their first course of cisplatin ≥60 mg/m^2^. For the MEC study, patients were required to be naive to MEC and HEC and scheduled to receive their first course of one or more of the following agents alone or in combination: intravenous cyclophosphamide (<1500 mg/m^2^), doxorubicin, epirubicin, carboplatin, idarubicin, ifosfamide, irinotecan, daunorubicin, and/or intravenous cytarabine (>1 g/m^2^). The study protocol prespecified that ≥50% of patients enrolled in the MEC study would receive AC‐based chemotherapy (now reclassified as HEC).^6^

### 2.2 Treatment

In all 3 trials, patients were administered 180 mg oral rolapitant or placebo on day 1, approximately 1‐2 hours before chemotherapy. In the HEC trials, patients also received 10 μg/kg intravenous granisetron and 20 mg oral dexamethasone before chemotherapy on day 1 and 8 mg oral dexamethasone twice daily on days 2‐4.^18^ In the MEC trial, patients received 2 mg oral granisetron and 20 mg oral dexamethasone on day 1 and 2 mg oral granisetron on days 2 and 3.^19^

### 2.3 Assessment of nausea

Patients self‐assessed nausea each morning for 5 days following chemotherapy. To indicate the severity of nausea experienced during the preceding 24 hours, patients marked a VAS ranging from 0 to 100 mm. The percentages of patients with NN (maximum VAS <5 mm) and NSN (maximum VAS <25 mm) were calculated for the overall, delayed, and acute phases of CINV in cycle 1, as described previously.^11^, ^12^, ^18^, ^19^ The duration of nausea and significant nausea, measured by assessing the total number of days a patient experienced nausea (0‐5 days), was also evaluated.

### 2.4 Assessment of the impact of nausea on daily life

On day 6, patients self‐assessed the impact of CINV on daily life using the validated Functional Living Index‐Emesis (FLIE) questionnaire,^3^, ^21^ which contains 9 nausea‐related questions and 9 vomiting‐related questions. Responses to each question were scored on a 100‐mm, 7‐point VAS, higher scores corresponded to reduced impact of symptoms. Responses to the 9 nausea‐related questions were summed to calculate the nausea domain score (range, 9‐63).

### 2.5 Statistical analyses

Patients in the modified intention‐to‐treat population (those who received ≥1 dose of study drug at a GCP‐compliant site) were assessed for the efficacy endpoints of NSN and NN.^18^, ^19^ For these analyses, data were pooled across trials and grouped by type of chemotherapy administered (cisplatin‐based, carboplatin‐based, or AC‐based). Assessments of nausea and its impact on daily life were analyzed post hoc in the rolapitant and control groups. Between‐treatment‐group comparisons for efficacy binary endpoints were conducted using the Cochran‐Mantel‐Haenszel χ^2^ test, stratified for sex and study for the pooled HEC studies. Between‐treatment‐group comparisons for FLIE nausea domain scores were conducted using an analysis of variance model with sex and study (for the pooled HEC studies) as factors. *P* values <.05 were considered statistically significant. No adjustments for multiplicity were performed.

## 3 RESULTS

### 3.1 Patients

Overall, 1070 patients received cisplatin‐based chemotherapy, 703 received AC‐based chemotherapy, and 401 received carboplatin‐based chemotherapy (Table 1). The most common tumor types were lung cancer in the carboplatin‐based (52.1%) and cisplatin‐based (43.6%) chemotherapy groups and breast cancer in the AC‐based chemotherapy group (96.7%). Most patients receiving cisplatin‐based chemotherapy were male, whereas most receiving carboplatin‐based or AC‐based chemotherapy were female. The majority of patients were <65 years of age. Within each chemotherapy group, patient baseline characteristics were generally well balanced between the rolapitant and control arms (Table 1).

**Table 1:** Patient baseline characteristics by chemotherapy type

Characteristic	Cisplatin‐Based^18^		AC‐Based^19^		Carboplatin‐Based^22^	
	Rolapitant (n = 535)	Control (n = 535)	Rolapitant (n = 344)	Control (n = 359)	Rolapitant (n = 192)	Control (n = 209)
Age, y						
Median	59	59	54	53	61	64
Min, max	21, 86	18, 90	23, 86	22, 79	31, 83	23, 88
Age category, no. (%)						
<65 y	397 (74.2)	393 (73.5)	287 (83.4)	303 (84.4)	124 (64.6)	111 (53.1)
≥65 y	138 (25.8)	142 (26.5)	57 (16.6)	56 (15.6)	68 (35.4)	98 (46.9)
Sex, no. (%)						
Male	337 (63.0)	336 (62.8)	12 (3.5)	7 (1.9)	88 (45.8)	93 (44.5)
Female	198 (37.0)	199 (37.2)	332 (96.5)	352 (98.1)	104 (54.2)	116 (55.5)
Receipt of concomitant emetogenic chemotherapy, no. (%)^a^						
Yes	87 (16.3)	101 (18.9)	344 (100)	359 (100)	26 (13.5)	37 (17.7)
No	448 (83.7)	434 (81.1)	0 (0)	0 (0)	166 (86.5)	172 (82.3)

AC, anthracycline/cyclophosphamide; HEC, highly emetogenic chemotherapy; MEC, moderately emetogenic chemotherapy.

a Hesketh level ≥3.^25^

### 3.2 Nausea assessments

Nausea results from the individual trials have previously been published.^18^, ^19^ In patients receiving cisplatin‐based chemotherapy, significantly more patients receiving rolapitant than control reported NN in the overall, delayed, and acute phases (Table 2).^18^ In addition, a significantly higher percentage of patients reported NSN with rolapitant than with control in the overall, delayed, and acute phases (Table 3).^18^ Furthermore, patients receiving rolapitant experienced nausea for a shorter duration than those receiving control (Table 4).

**Table 2:** Patients with NN (maximum VAS <5 mm on a 0‐100 mm scale) by chemotherapy type and CINV phase

	Rolapitant		Control		Absolute Benefit, %^a^	*P* b
	No.	NN, %	No.	NN, %		
Overall phase (0‐120 h)						
Cisplatin‐based^18^	535	52.3	535	41.7	10.6	<.001
Carboplatin‐based^22^	192	62.5	209	51.2	11.3	.023
AC‐based^19^	344	34.9	359	36.2	−1.3	.713
Delayed phase (>24‐120 h)						
Cisplatin‐based^18^	535	55.7	535	44.3	11.4	<.001
Carboplatin‐based^22^	192	64.1	209	53.6	10.5	.034
AC‐based^19^	344	38.1	359	39.8	−1.7	.634
Acute phase (≤24 h)						
Cisplatin‐based^18^	535	70.5	535	64.3	6.2	.030
Carboplatin‐based^22^	192	80.7	209	77.0	3.7	.366
AC‐based^19^	344	54.9	359	59.3	−4.4	.240

AC, anthracycline/cyclophosphamide; CINV, chemotherapy‐induced nausea and vomiting; HEC, highly emetogenic chemotherapy; MEC, moderately emetogenic chemotherapy; NN, no nausea; VAS, visual analog scale.

a % difference (rolapitant minus control).

b *P* values obtained from the Cochran‐Mantel‐Haenszel χ^2^ test, stratified for study and sex for the pooled HEC studies.

**Table 3:** Patients with NSN (maximum VAS <25 mm on a 0‐100 mm scale) by chemotherapy type and CINV phase

	Rolapitant		Control		Absolute Benefit, %^a^	*P* b
	No.	NSN, %	No.	NSN, %		
Overall phase (0‐120 h)						
Cisplatin‐based^18^	535	72.1	535	65.4	6.7	.017
Carboplatin‐based^22^	192	80.7	209	72.7	8.0	.059
AC‐based^19^	344	63.7	359	62.4	1.3	.728
Delayed phase (>24‐120 h)						
Cisplatin‐based^18^	535	74.0	535	66.9	7.1	.011
Carboplatin‐based^22^	192	82.3	209	74.2	8.1	.050
AC‐based^19^	344	66.6	359	66.0	0.6	.877
Acute phase (≤24 h)						
Cisplatin‐based^18^	535	88.2	535	82.6	5.6	.009
Carboplatin‐based^22^	192	90.6	209	91.4	−0.8	.790
AC‐based^19^	344	75.9	359	79.7	−3.8	.227

AC, anthracycline/cyclophosphamide; CINV, chemotherapy‐induced nausea and vomiting; HEC, highly emetogenic chemotherapy; MEC, moderately emetogenic chemotherapy; NSN, no significant nausea; VAS, visual analog scale.

a % difference (NSN with rolapitant minus NSN with control).

b *P* values obtained from the Cochran‐Mantel‐Haenszel χ^2^ test, stratified for study and sex for the pooled HEC studies.

**Table 4:** Number of days with NN (VAS <5 mm on a 0‐100 mm scale) and NSN (VAS <25 on a 0‐100 mm scale) during the first 5 d after chemotherapy

Chemotherapy type	No. of days	NN, %		NSN, %	
		Rolapitant	Control	Rolapitant	Control
Cisplatin‐based		n = 535	n = 535	n = 535	n = 535
	0‐3	38.3	47.7	19.8	26.5
	4‐5	61.7	52.3	80.2	73.5
Carboplatin‐based		n = 192	n = 209	n = 192	n = 209
	0‐3	28.1	37.8	13.0	18.7
	4‐5	71.9	62.2	87.0	81.3
AC‐based		n = 344	n = 359	n = 344	n = 359
	0‐3	55.5	53.2	27.9	29.0
	4‐5	44.5	46.8	72.1	71.0

AC, anthracycline/cyclophosphamide; NN, no nausea; NSN, no significant nausea; VAS, visual analog scale.

In patients receiving carboplatin‐based chemotherapy, a significantly higher percentage of patients treated with rolapitant than with control reported NN during the overall and delayed phases (Table 2).^22^ A higher percentage of carboplatin‐treated patients receiving rolapitant than control also reported NSN in the overall and delayed phases (Table 3).

Among patients receiving AC‐based chemotherapy, NN and NSN rates were similar in patients receiving rolapitant or control (Tables 2 and 3). In addition, rates of NN and NSN for the 4‐5 days of chemotherapy were similar in patients receiving rolapitant or control (Table 4).

Rescue medication may mask nausea symptoms, which could preclude accurate evaluation of the efficacy of nausea prevention. Therefore, we sought to assess nausea in the absence of RM. Regardless of the type of chemotherapy, results for the endpoints of NN and no RM or NSN and no RM were similar to results for endpoints that did not assess RM use in all CINV phases. For patients receiving cisplatin‐based chemotherapy, rolapitant vs control rates of NN and no RM were overall, 51.0% vs 41.1% (*P *=* *.001); delayed, 54.6% vs 43.6% (*P *<* *.001); and acute, 69.0% vs 63.0% (*P *=* *.038); rolapitant vs control rates of NSN and no RM were overall, 67.7% vs 60.2% (*P *=* *.011); delayed, 69.9% vs 61.7% (*P *=* *.005); and acute, 85.6% vs 78.5% (*P *=* *.002). For patients receiving carboplatin‐based chemotherapy, rolapitant vs control rates of NN and no RM were overall, 60.4% vs 48.3% (*P *=* *.015); delayed, 63.0% vs 51.2% (*P *=* *.017); and acute, 79.7% vs 75.6% (*P *=* *.327); rolapitant vs control rates of NSN and no RM were overall, 74.5% vs 65.1% (*P *=* *.041); delayed, 76.6% vs 66.0% (*P *=* *.020); and acute, 89.1% vs 87.1% (*P *=* *.542). For patients receiving AC‐based chemotherapy, rolapitant vs control rates of NN and no RM were overall, 34.0% vs 34.8% (*P *=* *.822); delayed, 37.2% vs 37.6% (*P *=* *.914); and acute, 54.4% vs 57.9% (*P *=* *.339); rolapitant vs control rates of NSN and no RM were overall, 58.1% vs 56.5% (*P *=* *.670); delayed, 61.3% vs 59.1%, (*P *=* *.537); and acute, 72.1% vs 75.8% (*P *=* *.268).

### 3.3 FLIE nausea domain scores

Improvements in nausea domain scores with rolapitant compared with control were statistically significant in the cisplatin‐based chemotherapy group but not in the carboplatin‐based chemotherapy group (Table 5). Mean nausea domain scores were similar with rolapitant and control in the AC‐based chemotherapy groups.

**Table 5:** FLIE nausea domain scores^a^ by chemotherapy type

Chemotherapy Type	Rolapitant		Control		Mean Difference (95% CI)^b^	*P* c
	No.	Mean (SD)	No.	Mean (SD)		
Cisplatin‐based	493	55.3 (11.3)	480	53.5 (13.5)	1.8 (0.2‐3.4)	.020
Carboplatin‐based	180	57.9 (9.7)	189	55.6 (12.4)	2.3 (0.0‐4.6)	.051
AC‐based	315	51.2 (13.6)	328	50.2 (14.3)	1.0 (−1.2‐3.2)	.440

AC, anthracycline/cyclophosphamide; ANOVA, analysis of variance; CI, confidence interval; CINV, chemotherapy‐induced nausea and vomiting; FLIE, Functional Living Index‐Emesis; HEC, highly emetogenic chemotherapy; MEC, moderately emetogenic chemotherapy; SD, standard deviation.

a Range, 9‐63.

b Rolapitant vs control.

c For the pooled HEC studies, *P* values were obtained using an ANOVA model adjusting for study and sex; for the MEC study, *P* values were obtained adjusting for sex.

## 4 DISCUSSION

Whereas the addition of NK‐1RAs to the combination of 5‐HT_3_ RA and dexamethasone has significantly reduced rates of emesis in CINV, control of nausea, especially in the delayed phase, remains a clinical challenge.^13^, ^14^, ^15^, ^16^, ^23^ Given the subjective nature of measuring nausea,^17^ patients may find it easier to report the presence or absence of nausea (using NN) rather than the varying degrees of nausea (using NSN), rendering NN a more quantifiable endpoint than NSN.^14^, ^16^ In this analysis, rolapitant significantly improved NN rates during the delayed and overall phases in patients receiving carboplatin‐based chemotherapy. In addition, rolapitant significantly improved both NN and NSN rates during all phases in patients receiving cisplatin‐based chemotherapy. These results demonstrate that the addition of rolapitant to a 5‐HT_3_ RA and dexamethasone regimen has a meaningful effect in reducing the incidence of nausea in these populations. These results are important as patients receiving chemotherapy view nausea as their highest concern.

Due to the subjective nature of quantifying nausea, nausea is inconsistently reported in the literature. In studies of NK‐1RAs in patients receiving cisplatin‐based chemotherapy that have assessed and reported nausea as an exploratory endpoint, the findings have been inconsistent.^18^, ^24^, ^25^, ^26^, ^27^, ^28^, ^29^

Although rolapitant significantly reduced the rates of NN and NSN in patients receiving cisplatin, approximately one‐half of the patients receiving rolapitant in this study reported nausea, and approximately one‐quarter reported significant nausea. Olanzapine is another antiemetic agent, which can be combined with an antiemetic triple regimen (NK‐1 RA, 5‐HT_3_ RA and dexamethasone) or used as a rescue medication for breakthrough CINV. A randomized, double‐blinded phase 3 trial of 380 patients treated with HEC evaluated the benefit of adding olanzapine to a triple regimen of an NK‐1 RA, a 5‐HT_3_ RA, and dexamethasone.^2^ The primary endpoint of this trial was nausea prevention. Patients receiving olanzapine showed significant improvement in rates of NN for the acute, delayed, and overall phases compared with patients receiving control,^2^ demonstrating the effectiveness of combining olanzapine with a 3‐drug antiemetic regimen containing an NK‐1RA. Consistent with previous studies, the incidence of NN was lower in the delayed and overall phases than in the acute phase.

In the current analysis, patients receiving cisplatin‐based or carboplatin‐based chemotherapy reported lower rates of nausea than those receiving AC‐based chemotherapy, consistent with other studies of NK‐1RAs.^30^, ^31^, ^32^ Factors contributing to the higher rates of nausea in AC‐based chemotherapy include the impact of high emetogenicity, the higher proportion of patients <65 years, and the higher proportion of females, which are also all risk factors for CINV.^33^

Rolapitant provides effective control of nausea, as indicated by improved NN and NSN rates in patients receiving cisplatin‐based or carboplatin‐based chemotherapy. Based on the post hoc analyses presented herein, the clinical benefit of rolapitant administration was similar regardless of RM use, indicating that use of RM did not confound the analysis of nausea, likely because RMs were not intended to be used prophylactically in this study. Prospective randomized control trials should be conducted to confirm these results. In addition to lowering the frequency of nausea, reducing the duration of nausea is clinically relevant. An exploratory analysis assessed days of NN, a novel way to evaluate nausea duration (Table 4). Given the negative impact of nausea on QoL,^5^ assessments should focus not only on the frequency of nausea but also on intensity and duration using the quantifiable endpoints of NN and total number of days of NN.

Given the unmet need to reduce nausea incidence after chemotherapy and the future emphasis on nausea prevention in CINV management within the NK‐1 RA class, this analysis supports the effectiveness of rolapitant for the prevention of nausea during the delayed and overall phases in patients receiving cisplatin‐ or carboplatin‐based chemotherapy.

## CONFLICT OF INTEREST

Rudolph M. Navari reports consulting fees for TESARO, Inc. Bernardo Rapoport reports grants, personal fees, and serving on advisory boards and speaker bureaus for TESARO, Inc.; personal fees and other from MSD; and personal fees from Herron. Dan Powers and Sujata Arora are employees of TESARO, Inc. and may own stock in the company. Rebecca Clark‐Snow reports serving on an advisory board and a speaker bureau for Merck.
